# GABAergic interneuron diversity and organization are crucial for the generation of human-specific functional neural networks in cerebral organoids

**DOI:** 10.3389/fncel.2024.1389335

**Published:** 2024-04-11

**Authors:** Sebastian H. Heesen, Georg Köhr

**Affiliations:** ^1^Molecular and Behavioural Neurobiology, Department of Psychiatry and Psychotherapy, University Hospital, Ludwig Maximilian University of Munich, Munich, Germany; ^2^Department of Neurophysiology, Mannheim Center for Translational Neurosciences, Heidelberg University, Mannheim, Germany; ^3^Physiology of Neural Networks, Central Institute of Mental Health, Heidelberg University, Mannheim, Germany

**Keywords:** human-specific inhibition, GABAergic interneurons, E-I balance, phase-amplitude-coupling, functional neural networks, 2D/3D neuronal cell culture, cerebral organoids

## Abstract

This mini review investigates the importance of GABAergic interneurons for the network function of human-induced pluripotent stem cells (hiPSC)-derived brain organoids. The presented evidence suggests that the abundance, diversity and three-dimensional cortical organization of GABAergic interneurons are the primary elements responsible for the creation of synchronous neuronal firing patterns. Without intricate inhibition, coupled oscillatory patterns cannot reach a sufficient complexity to transfer spatiotemporal information constituting physiological network function. Furthermore, human-specific brain network function seems to be mediated by a more complex and interconnected inhibitory structure that remains developmentally flexible for a longer period when compared to rodents. This suggests that several characteristics of human brain networks cannot be captured by rodent models, emphasizing the need for model systems like organoids that adequately mimic physiological human brain function *in vitro*.

## 1 Introduction

The necessity to understand the functional properties of neural networks beyond the mere characterization of their anatomical structure and existence of neural projections is rapidly rising. This surge is driven by the growing scientific desire to create accurate *in vitro* representations of human brain regions and pathologies. Current hiPSC-derived two-dimensional (2D) cell cultures are argued to poorly imitate crucial functional characteristics of human brain networks (Centeno et al., 2018). Over the past few decades, the balance between inhibition and excitation (E-I balance) has been linked to many neurological diseases (Rubenstein and Merzenich, 2003; Spencer et al., 2003; Scharfman, 2007; Ferguson and Gao, 2018). Evolutionarily speaking the diversity in inhibitory signaling appears to strongly determine the cerebral and cognitive complexity of an organism. This review will examine evidence suggesting that an inaccurate representation of the inhibitory and excitatory network components is the main culprit of unsatisfactory network modeling. Moreover, it aims to identify whether diversity of inhibition might be the defining property that could render brain organoids functional model systems to mimic human brain function, thereby shedding light on the complex and species-specific effects that inhibition exerts onto physiological human brain networks.

## 2 Cerebral organoids

Typical human-derived neuronal 2D culture models have certain limitations such as a lack of glial cell support and their exclusively planar neurite outgrowth which deviates far from realistic growth behavior and potentially affects functionality to a great extent (Poli et al., 2019). Cerebral organoids represent three-dimensional (3D) neuronal tissue that strives to simulate the functional connectivity between brain regions. They are derived from pluripotent stem cells and self-organize into mini-organ-like structures in rotating bioreactors over several months (Passaro and Stice, 2021; Sun et al., 2021). Brain organoids aim to recapitulate defined developmental stages and replicate existing circuits. Consequently, brain organoids are often reasoned to represent the missing link between 2D cell cultures and *in vivo* models (Centeno et al., 2018). Translational and basic research as well as the field of personalized medicine hope to benefit from physiologically relevant 3D models for an improved understanding of many neurological disorders, risk factors and possible cures.

## 3 Functional neural networks

The functionality of a neural network can be defined as its ability to successfully process information that is specific to the corresponding network. The precise timing of synchronized action potentials in the correct locations is regarded as the underlying mechanism of this spatiotemporal encoding (Singer, 1993; Cobb et al., 1995). When assessing a neural network, a first indicator for its functionality is a healthy morphological appearance. This includes dendritic density and outgrowth as well as the presence of short- and long-distance cross-linkages (Stam and van Straaten, 2012; Lee et al., 2015). However, a defined morphological appearance does not prove the capacity of the network to propagate electrical signals mirroring physiological functionality. Hence, the most common analysis tools to investigate the functionality of neural networks of brain organoids are based on electrophysiology (Passaro and Stice, 2021). The functionality of individual neurons can be elucidated comparatively easily using whole-cell patch clamping in acute brain slices, even from human 3D cultures (Paşca et al., 2015). However, information about whole-network connectivity or global functionality can thereby not be obtained. Therefore, indirect approaches for the quantification of whole-network dynamics have been developed such as all-optical electrophysiology whereby fluorescent markers indicate ionic fluxes during depolarization events (Kiskinis et al., 2018) or imaging of synchronized transient calcium currents (Lancaster et al., 2013; Sakaguchi et al., 2019).

Direct electrical measurements of entire networks can be conducted using devices known as microelectrode arrays (MEAs) which contain a grid of a large number of microscopic electrodes that enable the capture of network activity in real-time over large regions of two-dimensional cell cultures (Passaro and Stice, 2021). However, the planar electrode arrays can only touch limited areas of the surface of a 3D organoid, thus the accessibility of inner neurons is greatly decreased. Passaro and Stice (2021) have outlined multiple solutions to address this issue, including inner regional recordings either by inserting probes or neuron-like electronics, or by placing mesh nanoelectronics into the center of developing organoids. Other similar strategies to overcome the issue entail e.g., pit-shaped arrays or 3D MEA systems where a large number of microelectrodes is integrated on different height levels of thin needle-like tools which were successfully inserted into a small engineered human iPSC-derived organoids (Shin et al., 2021). Recently, a kirigami-inspired electrophysiology platform was created that eliminates the need to plate suspended organoids, thereby preserving their self-organized 3D structure (Yang et al., 2024).

### 3.1 Brain waves and synchronous neuronal activity

The described electrophysiology-based whole-network approaches seek to determine whether a large neural network is healthy and functional by analyzing the emerging brain waves. Brain waves are oscillatory neural activity patterns that arise from synchronized activity of functional neuronal units. A fundamental trait of functional networks appears to be the capacity to embed small regional oscillations into the framework of global brain communication, thereby transferring different types of information across distant brain networks via oscillations. At the network level, this cross-frequency coupling is commonly achieved through the amplitude modulation of fast local oscillations by the phase of slow large-scale rhythms, e.g., coupling of slow global θ- or respiration-entrained rhythms to different specific fast γ-subbands (Zhong et al., 2017). This interaction is known as phase-amplitude-coupling (Tort et al., 2010).

Two mechanisms are thought to govern the entrainment of these synchronized brain rhythms: electrical coupling of neighboring cells via gap junctions is one of these mechanisms. The removal of functional gap junctions via genetic knockout of Connexin-36 in an investigated neuronal population has shown that sub-threshold neuronal oscillations ceased to be synchronized in the absence of gap junctions (Leznik and Llinás, 2005). Apart from gap junction-mediated cell-to-cell current fluxes, synaptic coupling between excitatory and inhibitory neurons leading to feedback inhibition is thought to be the major mechanism of synchronization. Many GABAergic cortical interneurons such as chandelier cells innervate numerous cortical pyramidal cells in a local area (Li et al., 1992). The discharge of a single interneuron can consequently hyperpolarize a population of pyramidal cells of over a thousand neurons at the same time and give rise to the synchronized oscillations that play a paramount role in defining a functional network (Cobb et al., 1995; Scharfman, 2007). A delicate E-I balance in a locally and temporally precisely regulated framework is therefore crucial for neural networks to be functional.

## 4 GABA-mediated network inhibition

Excitatory action in the mammalian central nervous system (CNS) is primarily mediated by the neurotransmitter glutamate (Dingledine et al., 1999). In the mature mammalian CNS, glutamate is opposed by γ-aminobutyric acid (GABA) which acts as the main inhibitory neurotransmitter (Costa, 1998). The recycling of these neurotransmitters is largely dependent on the presence and proper arrangement of presynaptic neurons and surrounding astrocytes. By removing most of the synaptic GABA and glutamate, converting both to glutamine and shuffling it back to the presynaptic neuron (glutamate/GABA-glutamine cycle), astrocytes are fundamental to maintaining the homeostasis of excitation and inhibition (Hertz, 2013; Andersen and Schousboe, 2023).

### 4.1 GABA receptor subtypes and the GABAergic polarity switch

GABA interacts with two major receptor subtypes: GABA_A_ receptors, ionotropic and selectively permeable to chloride ions (Cl^–^) and GABA_B_ receptors which are metabotropic, mediating a slow and sustained suppression of neuronal excitability.

The study of developing network inhibition is complicated by a phenomenon known as the GABAergic polarity switch. The first described hyperpolarizing (inhibitory) effects that GABA exerts onto mature neurons are contrasted by depolarizing (excitatory) effects on immature neurons (Ben-Ari et al., 2007). Mechanistically, the excitatory action of GABA is primarily explained by high intracellular Cl^–^ levels established by the prevailing expression of the Cl^–^ loader NKCC1 and the low expression of the Cl^–^ extruder KCC2. This GABAergic polarity switch persists in rodents 14–15 days after birth and is of particular interest for the modeling of developmental brain disorders (Kang et al., 2019). The switch has been observed in bioengineered neuronal organoids at a later time point, at day 40 (Zafeiriou et al., 2020), and could be especially relevant to the growth stages of brain organoids which are supposed to undergo a maturation process similar to neurons in a physiological environment.

### 4.2 Diversity of interneuron types

A great diversity of interneurons has been observed and is described to link different oscillatory patterns to each other in a timed and locally confined manner. This flexibly shapes neural activity into temporal patterns (Cardin, 2018; Kullander and Topolnik, 2021). Interneuron classifications remain a subject of ongoing discussion and vary depending on whether they are based on neurochemicals, function, morphology or developmental origin within the ganglionic eminences which lay out a path for the migration of glial and neuronal progenitor cells (Anderson et al., 2001; Encha-Razavi and Sonigo, 2003; Ghashghaei et al., 2007). A compelling framework segregates interneurons into three groups based on a combination of developmental origins and neurochemical occurrence: parvalbumin (PV), somatostatin (SST) and ionotropic serotonin receptor (5HT3aR)-expressing (Rudy et al., 2011; Pelkey et al., 2017; Tzilivaki et al., 2023).

(i) PV-expressing interneurons (including chandelier cells) are fast-spiking basket cells and the most abundant species (Galarreta and Hestrin, 2002; Puig et al., 2008; Rudy et al., 2011). Targeting cell somata, they regulate spiking probability of excitatory neurons through strong feedback inhibition on a short and precise timescale (Cruikshank et al., 2007; Cardin, 2018).
(ii) SST-expressing interneurons (including Martinotti cells) are dendrite-targeting and induce slight depression of synaptic inputs over an extended timescale upon repeated activation (Tan et al., 2008; Cardin, 2018).
(iii) 5HT3aR-expressing interneurons target dendrites of pyramidal neuron and SST-expressing interneurons (Cardin, 2018). Their functional role is the least understood among the three groups (Rudy et al., 2011). However, by regulating inhibitory neurons through disinhibition (Kullander and Topolnik, 2021), they present a potential direct mechanism of timing slow rhythms to superimposed fast local oscillations.

The three classes of interneurons have been shown to reciprocally interact with each other (Cardin, 2018; Tzilivaki et al., 2023) which creates timed neural-activity loops and gives rise to the complex coupling patterns of fast nested oscillations that occur inside large-scale rhythms. Notably, fast timescale nested oscillation inside slower rhythms have not been observed in 3D neurospheres suggesting that the necessary structure and diversity of glial and in particular inhibitory cell types might currently only be achieved in organoid models (Trujillo et al., 2019). The three groups of interneurons are present in varying distributions across the six layers of the neocortex. In rodents, layer 1 is almost exclusively populated by 5HT3aR-expressing interneurons, layers 4/5 are dominated by PV and layer 6 by SST (Rudy et al., 2011). This heterogenous physiological organization emphasizes the need for proper distribution of interneuron types to replicate the functional properties of specific cortical circuits. Cerebral organoids have been shown to form the six cortical layers *in vitro*. These layers also contain astrocytes, oligodendrocytes and other types of glial cells (Qian et al., 2020; Yang et al., 2022). Because of their involvement in neurotransmitter recycling, astrocytes are likely to influence strength and precision of synaptic transmission. Despite these general structural homologies of organoids to physiological networks, their lack of functional vascularization impedes efficient nutrient and oxygen distribution, typically resulting in a necrotic core (LaMontagne et al., 2022). Observed elevated metabolic stress that leads to constrained cell-type specification further exemplifies the limitations faced by current organoid technologies (Bhaduri et al., 2020).

### 4.3 GABAergic inhibition facilitates complex network function in organoids

**FIGURE 1:**
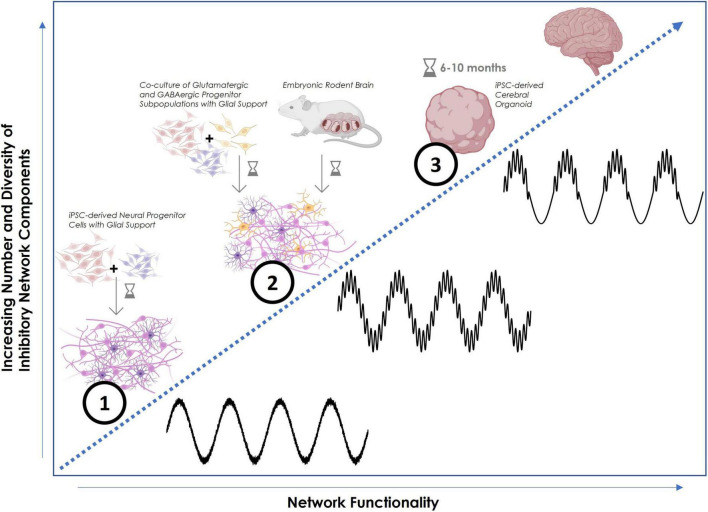
Schematic increase of network functionality with increasing complexity of inhibitory components. (1) Human neural progenitor cell (NPC)-derived neuronal cell culture, co-cultured with glial cells, showing basic repetitive oscillatory activity in the form of plain theta waves. The presence of astrocytes in particular helps to maintain E-I balance. (2) Co-culture of human NPC-derived neuronal cells, separately differentiated into inhibitory and excitatory subpopulations, co-cultured with glial cells. Spiking activity is more synchronous and begins to exhibit coherent dynamics in more balanced excitatory-inhibitory neural networks. Both theta and gamma oscillations occur independently or with minimal coupling. Comparable activity can be observed in a 2D primary neuronal cell culture prepared from embryonic rodent tissue containing a physiological variety of neuronal and supporting cell types. (3) Human iPSC-derived brain organoid (after 6–10 months of maturation) with multiple cortical layers and 3D structure mimicking authentic human brain regions that display higher order rhythms with cross-frequency coupling. These nested phase-amplitude-coupled oscillations are created by inhibitory feedback loops and are commonly part of local field potential (LFP) or EEG recordings *in vivo*. Faster local oscillations are encapsulated within broader global brain rhythms. This configuration facilitates the spatiotemporal encoding of information thus emulating genuine physiological brain function. In this example, gamma wave amplitude is controlled by the phase of the theta wave. Figure created using Biorender.com and Python.

Electrophysiological analysis of young organoids further revealed that the contained inner neural circuitry shows synchronized activity (Shin et al., 2021). Moreover, a small initial proportion of inhibitory neurons in the neuronal population after 6 months of cortical organoid development reached around 15% after 10 months (Trujillo et al., 2019). This increase in the fraction of inhibitory neurons correlated with an observed increase in network complexity, emphasizing the crucial role inhibitory neurons seem to play in the latter (Figure 1). Electrophysiological findings support the hypothesis that inhibitory interneurons are necessary and responsible for the creation of complex coupled oscillatory patterns that transfer the spatiotemporal information constituting physiological network function. To prove this, Samarasinghe et al. (2021) developed a fusion organoid platform in which ventral ganglionic eminence organoids were fused with cerebral cortical organoids. This approach introduced interneurons in addition to the excitatory connections, resulting in complex oscillatory patterns that were not observed in purely cortical organoids. This showed that the functional integration of interneurons is the determining factor for network complexity.

Comparison of patient electroencephalograms (EEGs) and patient-derived organoid readouts demonstrated a network complexity-functionality relationship. Minor interneuron composition variances in disease phenotype organoids may explain observed network irregularities and absence of specific oscillations in mutants.

These findings are strongly supported by a recent study that examined 3D horizontally bio-printed neuronal tissue (Yan et al., 2024). An increased synaptic density and frequency of spontaneous action potentials were found when GABAergic interneurons were included into the neuronal print compared to being omitted.

## 5 Consequences of inhibitory dysfunction

Temporal network organization is likely to be lost when inhibitory mechanisms malfunction (Cardin, 2018). In healthy brain networks, GABAergic interneurons put brakes on excitatory signaling–their loss has been linked to several neuropsychiatric disorders (Rubenstein and Merzenich, 2003; Petanjek et al., 2009; Ferguson and Gao, 2018). The most prominent example of inhibitory dysfunction is epilepsy. Epileptic seizures are thought to be the manifestation of hypersynchronous network activity in the absence of adequate inhibition. Excessive discharge alone can act as a major disruptor of E-I balance but tends to be insufficient to induce seizures. Consequently, epilepsy seems to only be fully characterized by uninhibited synchronous discharge across large brain regions (Scharfman, 2007). Forms of abnormal neural synchrony as observed in schizophrenia are another important example of inhibitory dysfunction. Phase locking and phase coherence associated to the binding of visual features were found to strongly deviate in schizophrenic patients (Spencer et al., 2003). This characteristic has possible implications for altered perception of reality, a frequent positive symptom of schizophrenia. Extending these clinical findings to organoid systems, abnormal activity resembling epilepsy has been shown to arise in organoids derived from Rett syndrome patient iPSCs (Samarasinghe et al., 2021). Interestingly, functional properties were partially restored by pharmacological intervention. This manifests both the possibility of creating functional replicas of human pathologies *in vitro* and the option to test the efficacy of drugs for restoring brain function.

## 6 Species-specific formation of E-I balance

### 6.1 Origins of neuronal progenitor cells

Prior investigations into the cortical neurodevelopment of rodents led to the establishment of a model in which excitatory and inhibitory cortical neurons exclusively originate from two distinct populations of progenitor cells (Gorski et al., 2002; Petanjek et al., 2009). According to this, excitatory glutamatergic neurons originate from progenitors located in the cortex (dorsal forebrain region) and inhibitory GABAergic interneurons originate exclusively from progenitors in the ganglionic eminences (ventral forebrain region) (Bandler et al., 2022). This observation in mice was assumed to hold true for humans as well. However, deviating from this, newer findings have unveiled that human cortical interneurons can additionally be formed from the same cortical progenitor cells which primarily generate excitatory neurons (Delgado et al., 2022). Human neuronal diversity and fate determination appear to remain modifiable deep into the developmental process. This can be speculated to enable more precise finetuning of inhibition demands for regional specifications and allow for developmental flexibility beyond the disappearance of the ganglionic eminences after around one year (Encha-Razavi and Sonigo, 2003). At this point the underlying molecular mechanisms that determine the cell fate and induce locally born inhibitory neurons are not understood (Delgado et al., 2022) which limits the possibility to recreate given mechanisms in organoid models.

### 6.2 Network complexity increases with increased number and diversity of GABAergic neurons

The location, number and diversity of GABAergic interneurons directly coordinate cortical function (Petanjek et al., 2009; Kullander and Topolnik, 2021). Looking at evolutionary evidence, a rise in network complexity has been observed to be accompanied by an increase in diversity of GABAergic neurons (Rakic, 2009; Vanderhaeghen and Polleux, 2023) suggesting an important role of inhibitory circuit diversity regulating functional complexity. Connectome and transcriptome analyses revealed a 2-3-fold higher proportion of interneurons in humans compared to rodents (Krienen et al., 2020; Bakken et al., 2021; Loomba et al., 2022). Surprisingly, despite this increase in inhibitory circuit elements, the synaptic input to pyramidal neurons and thereby the E-I balance only displays a very moderate shift toward increased inhibition. The human interneuron population was found to be primarily expanded toward bipolar-type interneurons (6–8 times; Loomba et al., 2022), favoring interneuron-to-interneuron circuits. In contrast, multipolar neurons, receiving substantially lower levels of inhibitory input, constitute the large majority in mice (Loomba et al., 2022). Thus, elaborated disinhibitory circuits in humans appear to give rise to unique network properties and functional complexity distinct from rodent brains.

Recent findings emphasize that due to the differences in network development and resulting network complexity, rodent-based models seem to inadequately reflect various features that define functional neural network activity in human brains. This serves as an explanation for the short-comings of many clinical trials when trying to transfer insights from rodent pathologies over to human diseases without additional testing on functional human cell-based assays (Samarasinghe et al., 2021; Zhang et al., 2023). Thus, the interspecies differences in organization, diversity and circuit integration of GABAergic interneurons are very likely to be the main driving force behind this lack of resemblance and transferability.

## 7 Conclusion

The electrophysiological analysis of brain organoids still poses a challenge to current research since most available assays are designed for 2D cultures. A number of new technologies are being developed and tested and will continue to make it more feasible to harness the full electrophysiological potential of organoid models. At present, 2D neuronal cell cultures are the most widely available, fastest and most robust environment *in vitro* to obtain data from functional brain networks. The 2D structure without cortical layers, however, is typically too limiting to allow for complex cross-frequency coupling of distinct oscillatory patterns. As replicated in brain organoids, the varying three-dimensional distribution of different GABAergic interneuron types across cortical layers and the arising inhibitory feedback loops can be hypothesized to play the primary role in giving rise to the complex nested brain waves that more closely characterize a physiological and truly functional network. Further limiting the transferability of primary neuronal rodent cell cultures to humans, structural differences in the number, diversity and interconnectivity of inhibitory network components between humans and rodents have been detected. Hence, to leverage insights into human-specific network activity patterns, the culturing of human-induced pluripotent stem cell-derived brain organoids will have to be refined and subsequent advances in electrophysiological measurement platforms will be necessary. In conclusion, the evidence strongly indicates that the number, diversity and organization of inhibitory network components are the main factors determining network complexity and functionality. Physiological replication of inhibitory components consequently renders organoid-based model systems extremely promising candidates for future drug screening assays and disease modeling. The potential to successfully bridge the gap from current human 2D cell cultures, that are often functionally limited or unspecific to humans, over to clinical trials is immense.

## Author contributions

SH: Data curation, Investigation, Software, Writing – original draft. GK: Conceptualization, Funding acquisition, Supervision, Writing – review & editing.
